# Supplementary figures and images for: Active probing to highlight approaching transitions to ictal states in coupled neural mass models
Source: PLoS Comput Biol. 2021 Jan 25;17(1):e1008377. doi: 10.1371/journal.pcbi.1008377 (PMC7861539; doi:10.1371/journal.pcbi.1008377)

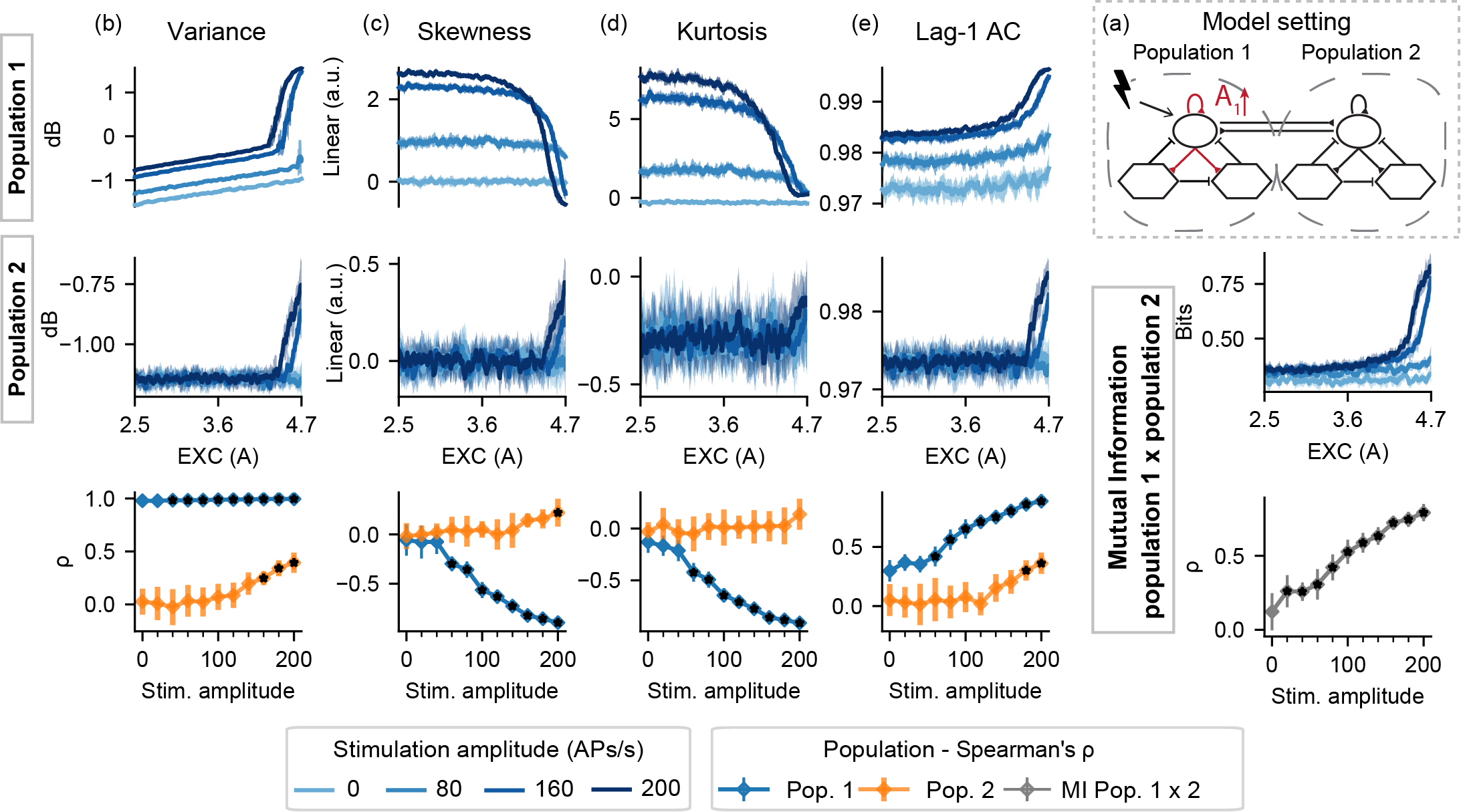

Supplement: S1 Fig — (a) shows a simplified illustration of the model setting. For (b) variance, (c) skewness, (d) kurtosis and (e) lag-1 AC features, top rows show feature values extracted from the output of each population, as the excitability gain parameter (A1) is increased. (f) shows the inter-population synchronization as measured by the mutual information feature. Bottom row shows Spearman’s rank correlation between each feature series and the shifted parameter (A1), as stimulus amplitude is increased. Black dots indicate significant differences from passive observation (stim. Amplitude = 0) according to Tukey’s HSD. For population 1, excitability increase (A1) is correlated with variance even with passive observation. Increased correlation of other features, including mutual information between population activities, with the ictogenic parameter A1 occurs only with stimulation. For features extracted from population 2, high stimulation amplitudes result in changes prior to ictal onset, even though this population was not stimulated–probably due to the ILS elicited in population 1. (TIF) [file pcbi.1008377.s001.tif]

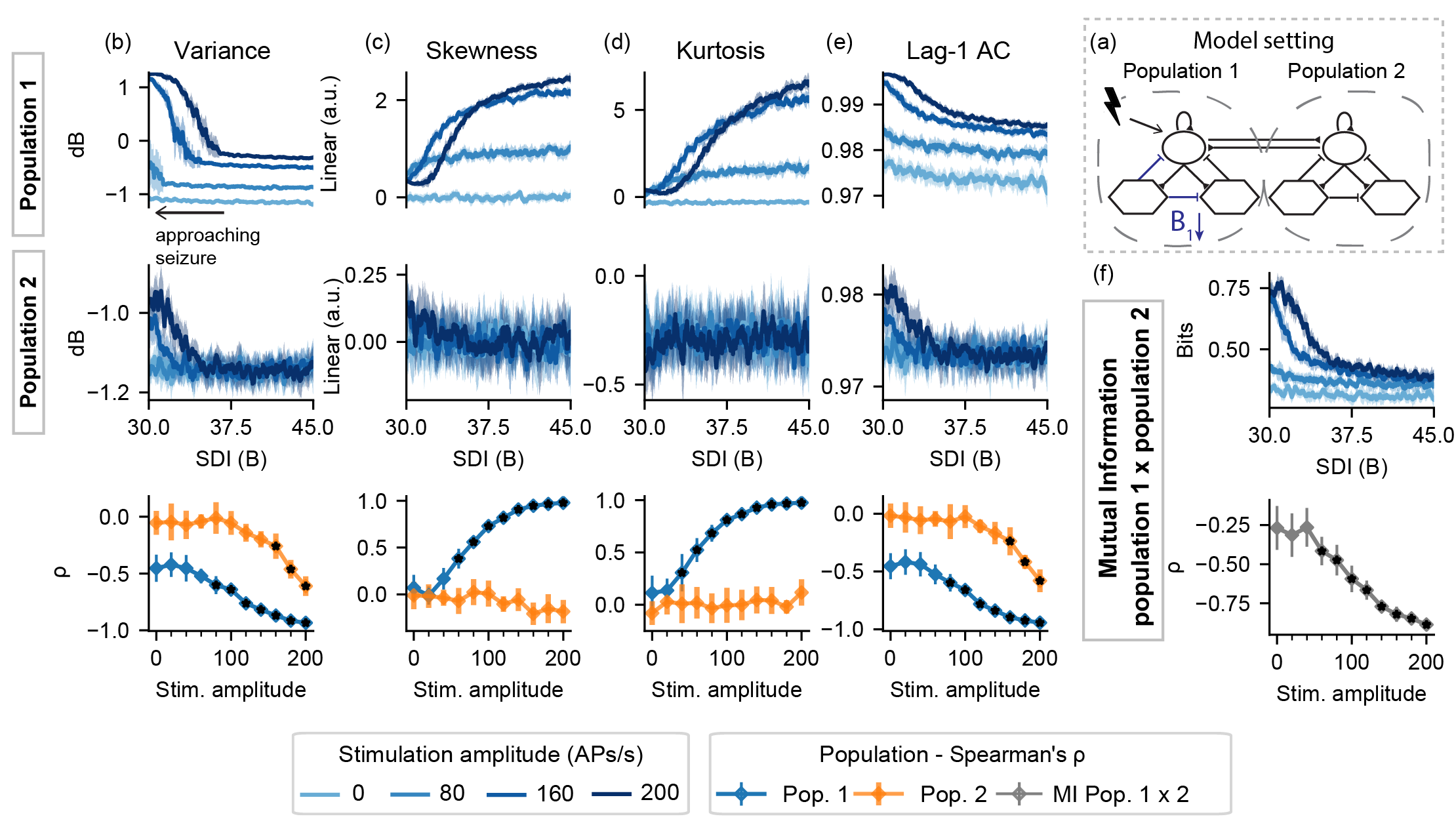

Supplement: S2 Fig — (a) shows a simplified illustration of the model setting. For (b) variance, (c) skewness, (d) kurtosis and (e) lag-1 AC features, top rows show feature values extracted from the output of each population, as the excitability gain parameter (A1) is increased. (f) shows the inter-population synchronization as measured by the mutual information feature. Bottom row shows Spearman’s rank correlation between each feature series and the shifted parameter (B1), as stimulus amplitude is increased. Black dots indicate significant differences from passive observation (stim. Amplitude = 0) according to Tukey’s HSD. For population 1, SDI decrease (B1) is slightly correlated with variance even with passive observation, but stimulation increases the correlation with this and other features, including mutual information between population activities. For features extracted from population 2, high stimulation amplitudes result in changes prior to ictal onset, even though this population was not stimulated–probably due to the ILS elicited in population 1. (TIF) [file pcbi.1008377.s002.tif]
