# Supplementary material for: Active probing to highlight approaching transitions to ictal states in coupled neural mass models
Source: PLoS Comput Biol. 2021 Jan 25;17(1):e1008377. doi: 10.1371/journal.pcbi.1008377 (PMC7861539; doi:10.1371/journal.pcbi.1008377)
Supplement: Attachment — Submitted filename: PCOMPBIOL2020_ResponseToReviewers.pdf [file pcbi.1008377.s003.pdf]

Corresponding Author:
Eduardo Mazoni Andrade Marçal Mendes
Departamento de Engenharia Eletrônica
Universidade Federal de Minas Gerais
Email: emmendes@cpdee.ufmg.br
Belo Horizonte, November 18 , 2020

Dear editors,

Regarding Ms. PCOMPBIOL-D-20-01787 **“Active probing to highlight approaching transitions to ictal states in coupled neural mass models”** by Vinícius Rezende Carvalho, Márcio Flávio Dutra Moraes, Sydney S. Cash, and Eduardo Mazoni Andrade Marçal Mendes: we have reviewed the Ms. and changed it according to the reviewers’ suggestions. Each point raised is addressed in the text below and accompanied by changes in the manuscript itself. Two files of the revised manuscript are attached; one with tracked changes and another is the final version with all changes applied. Two additional Figures were also added as “supporting information”.

We would like to take this opportunity to thank the reviewers and editors for the meticulous job. They raised important issues and suggestions that were addressed, substantially improving the quality of the submitted work.

Yours Sincerely

Eduardo Mendes

In black: Comments by the reviewers.

In blue: Responses to the issues raised by the editor and reviewers

In red: Changes made to the manuscript (lines refer to the marked-up version).

Reviewer #1: The article discusses the important issue of predicting epileptic discharges using the existing computational neural mass model. A feature of this work is the analysis of signals received from the model. The task of predicting discharges is solved by modeling external stimuli by changing the parameters of the model.

The disadvantages of the work include:

1. In the "abstract" section, authors should better identify the methods that were used in this particular work.

Response: We have specified the model used in the work and the target and rate of the stimulation, in addition to adapting some terms. We tried not to add an overwhelming amount of method details in order to stay within the word limits and keep the abstract clear and concise.

(abstract)

…This work evaluates the theoretical and mechanistic underpinnings of this strategy using two coupled populations of the well-studied Wendling neural mass model. Different model settings are evaluated, shifting parameters (excitability, slow inhibition, or inter-population coupling gains) from normal towards ictal states while probing stimuli are applied every 2 seconds to the input of either one or both populations. The correlation between the extracted features and the ictogenic parameter shifting indicates if the impending transition to the ictal state may be identified in advance. …

2. Using the term lag-1 autocorrelation without clarification at the initial mention.

Response: We clarified the meaning of the lag-1 autocorrelation measure.

(Section 1, lines 61-62)

lag-1 autocorrelation (which is the estimate of the correlation of a signal with a 1-sample delayed version of itself) [37,38]

3. Not all known mesoscale EEG models are listed on line 124 (for example

https://doi.org/10.1371/journal.pone.0114316, https://doi.org/10.1007/BF00202790, https://doi.org/10.1371/journal.pone.0239125).

Response: We thank the reviewer for pointing the new references - we were not aware of the work by Medvedeva et al.. We believe it will be interesting to discuss it in our research group.

Initially, we meant to highlight a few of the pioneering mesoscale models. But as was pointed out, it is worthwhile to cite other examples. Thus, we listed additional models.

(Section 2.1, lines 125-127)

Pioneering works on such mean-field (or lumped-parameter) models include those from Wilson & Cowan [68], Freeman [69], Lopes da Silva et al. [70], and Wright & Liley [71]. Other models of this kind have been used to study spike-wave (SW) seizures [72] and their abatement using single-pulse stimulation [73].

4. On line 229, it is not entirely clear what is "a moving average filter of size 20".

Response: It refers to a moving average (moving mean, or rolling mean) filter, commonly used in digital signal processing to smooth noisy time series. The size 20 referred to the order of the filter – that is, each filtered value is taken as the mean of the 20 neighbouring samples. In the text, the phrase was rewritten for clarity.

(Section 2.4, line 223-225)

A moving average filter of order 20 was used for smoothing the resulting feature time series.

5. Line 253. Please clarify what does "5 au" mean.

Response: It means "arbitrary units", which we initially considered to be the model's output units. However, the correct units should actually be mV (as used by https://doi.org/10.1103/PhysRevE.78.051917 and https://doi.org/10.1155/2016/3628247). This has been changed accordingly in the text and in Figure 2.

(Section 2.4, line 249)

Spikes were detected if a peak was found in the simulated signal with amplitude greater than 5 mV.

(Figure 2 is shown in Comment #6 of Reviewer 2)

5. In my opinion, the "Conclusions" section should be written in a little more detail.

Response: We have included additional methodological details and emphasized the contributions of our work.

(Section 5)

(line 484)

…As parameters of a neuronal population were shifted from normal towards ictal activity, responses showed changes that were in general not visible with the passive observation of the populations' activity, such as increased variance, lag-1 autocorrelation and synchrony between neuronal populations' activities….

(lines 491-493)

… This is often related to the loss of a systems' resilience and are common hallmarks of an impending critical transition…

(Lines 495-496)

Altogether, results indicate that low-frequency probing stimuli can provide predictive value and reveal underlying dynamics involved in transitions from normal to ictal states.

Reviewer #1: It should be emphasized that new was obtained by analyzing the signals of the existing Wendling model. In general, the work is done and described at a good level, the results are presented clearly and in sufficient detail.

Response: We thank the reviewer for the comments. Relevant issues were pointed out and we believe that the quality of the work has significantly improved by addressing these issues.

Reviewer #2: The Ms describes application of neural mass model to study stimulation-based assessment of system closeness to seizure. The stimulation-based biomarkers of approaching transitions are based on a critical slowing down signal measures and are shown be able to identify proximity of the ictal state. The study is original and offers useful, conceptual insight into the problem of seizure prediction using active paradigms. The authors are familiar with the literature relevant to the problem and describe their work in this context. In general it is very good work that deserves a publication. I have only minor comments to improve the Ms.

Response: The reviewer has demonstrated an excellent understanding of the work and we greatly appreciate the comments. Relevant and accurate remarks were presented on how to improve it, in addition to pointing out misplaced and missing references and typing mistakes that were overlooked by us.

Minor points:

1. The authors use 2 model settings – with unilateral and bilateral stimulation. The unilateral stimulation is delivered only to the side with the 'normal' set of parameters. It seems counterintuitive. The most direct test would be to selectively stimulate the population with 'pathological' set of parameters to see if the probing stimulus can identify the on-going process of ictogenesis. Such test is not performed. Please explain your rationale of probing 'normal' network rather than 'epileptic' one in the unilateral setting.

Response: The reviewer raises an excellent point, which we had extensively discussed before deciding to use the listed model settings. The rationale for unilateral stimulation of the 'normal' population is that we were looking for the 'unexpected' result. That is, we expected the on-going process of ictogenesis from population 1 to be highlighted by direct probing (and this is indeed the case, as shown in the additional figure), but unsure if indirect (applied to the 'normal' population) would have any predictive value – and this seems to be the case for increasing excitability, but not slow inhibition. Both strategies are interesting to evaluate in this model, as they might give insights on which conditions (in terms of stimulation target and recording site in relation to the epileptogenic zone) can an active probing approach provide predictive value. Furthermore, this may tell us something if and how this strategy can be adapted with local and remote closed-loop seizure suppression devices. Thus, we have included figures for unilateral stimulation of the ictogenic population as Supporting information.

We would also like to highlight that other model settings and stimulation parameters can be tested straightforwardly with the code available at https://github.com/vrcarva/WNMM_probing.

(Section 2.3, lines 208,209)

Unilateral probing of the ictogenic population 1 is evaluated in additional model settings, included in S1 and S2 Figs.

(Section 3.1, lines 321-324)

However, if unilateral stimulation is applied to the ictogenic population, as shown in S1 and S2 Figs, increased feature changes can be detected in both populations.

(Added supplementary figures)

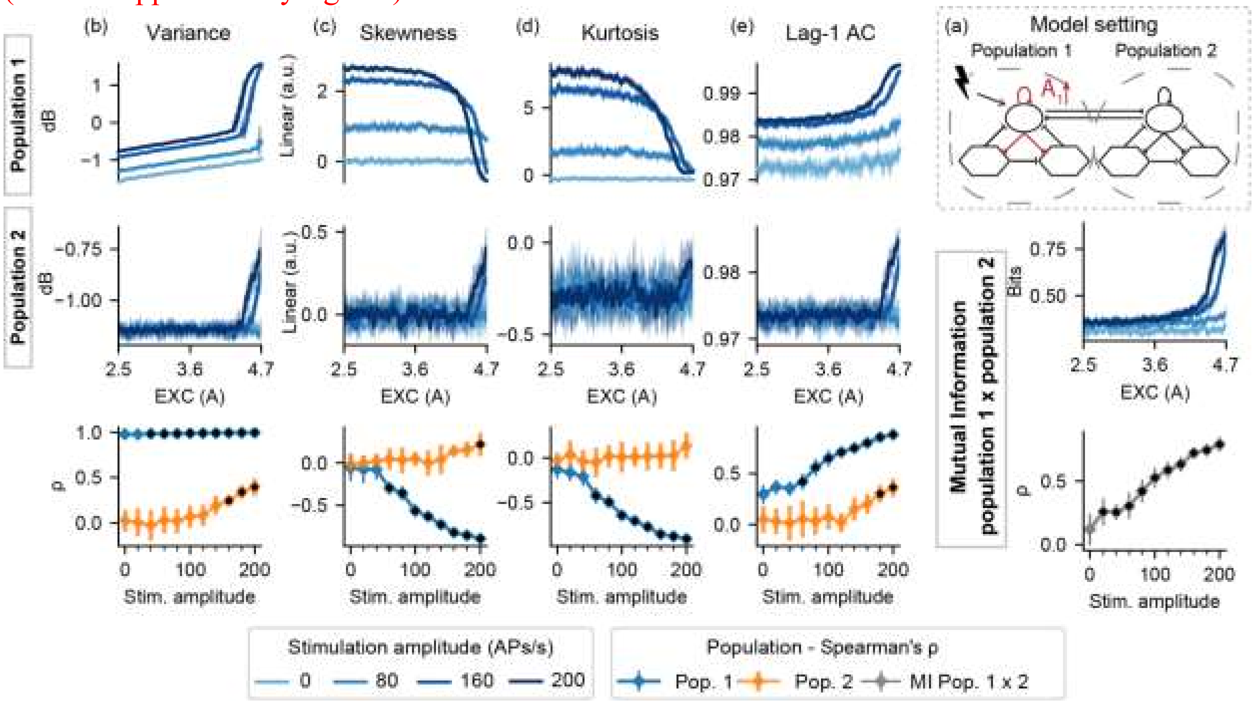

S1 Fig: **Feature series and probing efficiency for unilateral probing of ictogenic population with increased excitability.** (a) shows a simplified illustration of the model setting. For (b) variance, (c) skewness, (d) kurtosis and (e) lag-1 AC features, top rows show feature values extracted from the output of each population, as the excitability gain parameter (A_1) is increased. (f) shows the inter-population synchronization as measured by the mutual information feature. Bottom row shows Spearman's rank correlation between each feature series and the shifted parameter (A_1), as stimulus amplitude is increased. Black dots indicate significant differences from passive observation (stim. Amplitude = 0) according to Tukey's HSD. For population 1, excitability increase (A_1) is correlated with variance even with passive observation. Increased correlation of other features, including mutual information between population activities, with the ictogenic parameter A_1 occurs only with stimulation. For features extracted from population 2, high stimulation amplitudes result in changes prior to ictal onset, even though this population was not stimulated – probably due to the ILS elicited in population 1.

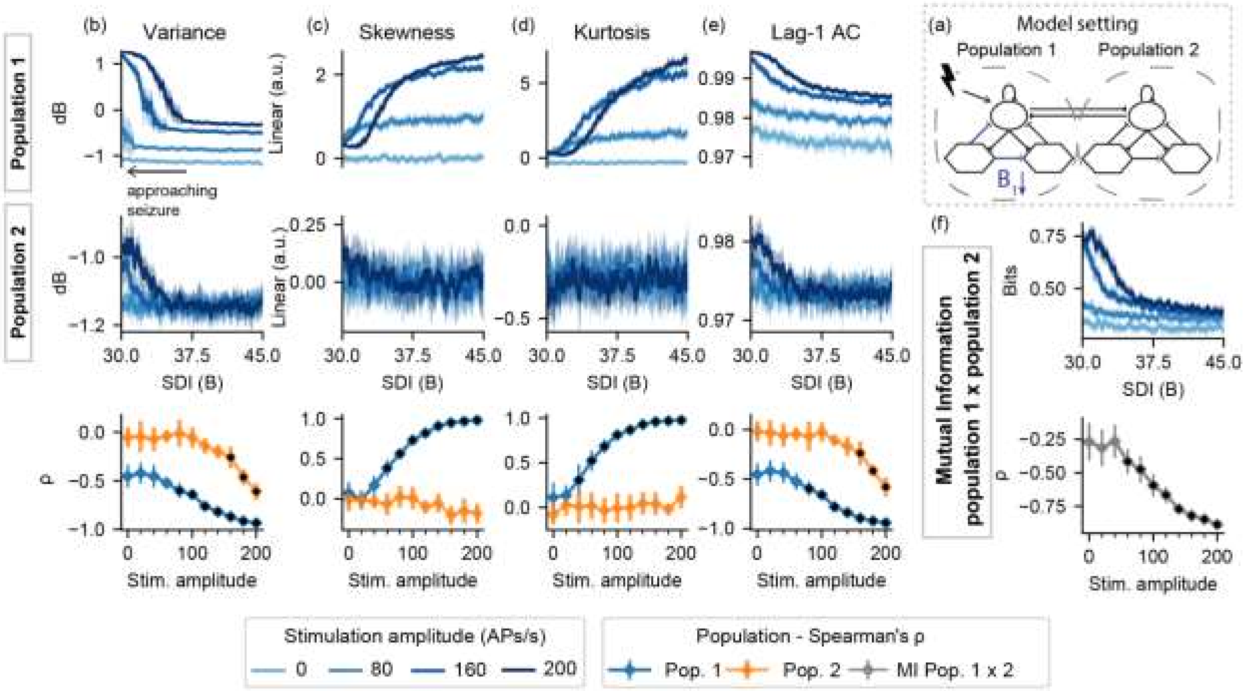

S2 Fig: **Feature series and probing efficiency for unilateral probing of ictogenic population with decreased slow inhibition.** (a) shows a simplified illustration of the model setting. For (b) variance, (c) skewness, (d) kurtosis and (e) lag-1 AC features, top rows show feature values extracted from the output of each population, as the excitability gain parameter (A_1) is increased. (f) shows the inter-population synchronization as measured by the mutual information feature. Bottom row shows Spearman's rank correlation between each feature series and the shifted parameter (B_1), as stimulus amplitude is increased. Black dots indicate significant differences from passive observation (stim. Amplitude = 0) according to Tukey's HSD. For population 1, SDI decrease (B_1) is slightly correlated with variance even with passive observation, but stimulation increases the correlation with this and other features, including mutual information between population activities. For features extracted from population 2, high stimulation amplitudes result in changes prior to ictal onset, even though this population was not stimulated – probably due to the ILS elicited in population 1.

2. Figure 4, top row, shows that Variance, Lag-1 AC and to some extend Kurtosis measured in Population 1 are sensitive to B parameter change (I-B setting). In the text it is described that feature changes indicating change of B were not found except AC(1). Please clarify.

Response: This is now corrected both in the results and the discussion sections, indicating that

variance and kurtosis have slight trends as B_1 is decreased.

(Section 3.1, Figure 4 caption)

Figure 4 shows slight changes in Lag-1 AC and variance (and to a lesser extent, in kurtosis as well) of population 1, regardless of stimulation. That is, as the SDI gain is decreased, no predictive effects of active probing are observed in the activity of the neuronal populations, and neither in the mutual information between both simulated signals.

(Section 4, lines 393-400)

In model setting I, extracted features (namely variance and lag-1 AC) from the ictogenic population 1 are slightly sensitive to the decrease of slow inhibition (B_1) and very sensitive to the increase in excitability (A_1), even with passive observation. For features extracted from the probed population 2, the effects depended on which parameter was being altered. No detectable changes were found in setting I-B, but increases in the coupling factor (K) and excitability (A_1) were correlated with features extracted from the perturbed population 2.

3. The meaning of black dots in Spearman rank correlations in all figures is not explained.

Response: The black dots indicate significantly different values (according to Tukey's honestly significant difference test), compared with the 'control' simulations (stimulation amplitude = 0). This is now clarified in-text and in the figure legend.

(Section 3.1, lines 287-288)

Black dots indicate significant differences from passive observation (stim. Amplitude = 0) according to Tukey's HSD.

4. Figures 4, 5, 7, 8 are not referred in the text.

Response: We thank you for pointing this out. Figures are now referred in the corresponding paragraphs in the "Results" section.

Lines 303 (Figure 4), 318 (Figure 5), 327 (Figure 6), 352 (Figure 7)

5. Model settings are named Ia, Ib etc (ln. 205-212). They are referred to as I-A, I-B later in the text. Please unify.

Response: Lines 205-212 were changed and the naming of model settings is now unified. Another mistake was corrected: "I.Probing neuronal population 1" should be "I.Probing neuronal

population 2”

(Lines 198-205)

I. **Probing neuronal population 2**

A. Increasing A_1: $A_1 = 2.5$ to 4.6, $A_2 = 4.0$, B = 40, K = 0.3

B. Decreasing B_1: A = 4.0, $B_1 = 45$ to 30, $B_2 = 40$, K = 0.3

K. Increasing K: A = 4.0, B = 40, K = 0 to 0.5

II. **Probing neuronal populations 1 and 2**

A. Increasing A_1: $A_1 = 2.5$ to 4.6, $A_2 = 4.0$, B = 40, K = 0.3

B. Decreasing B_1: A = 4.0, $B_1 = 45$ to 30, $B_2 = 40$, K = 0.3

K. Increasing K: A = 4.0, B = 40, K = 0 to 0.5

6. Figure 2 is a bit confusing, partly because of panel annotations a.1, a.2, b.1 etc. I suggest to mark the panels with headings e.g. ‘Population 1’, ‘Population 2’, ‘Active’, ‘Passive’ etc. It will help to quickly get information from the figure without going through the (complex) legend. Also the role of enlarged signals is not clear. What is the reader supposed to learn from them? I suggest to simplify the figure by removing them or at least making them smaller.

Response: We thank the reviewer for the suggestion. Indeed, marking panels with the suggested headings makes the figure much clearer, so we have modified it.

The enlarged figures should convey the shape and scale of the different types of activity (so we selected 2-3 segments) and response to stimuli of the simulated signals (with C showing the gradual amplitude and shape changes of the response). But they really take too much space, so we scaled these down on the vertical axis.

(Figure 2)

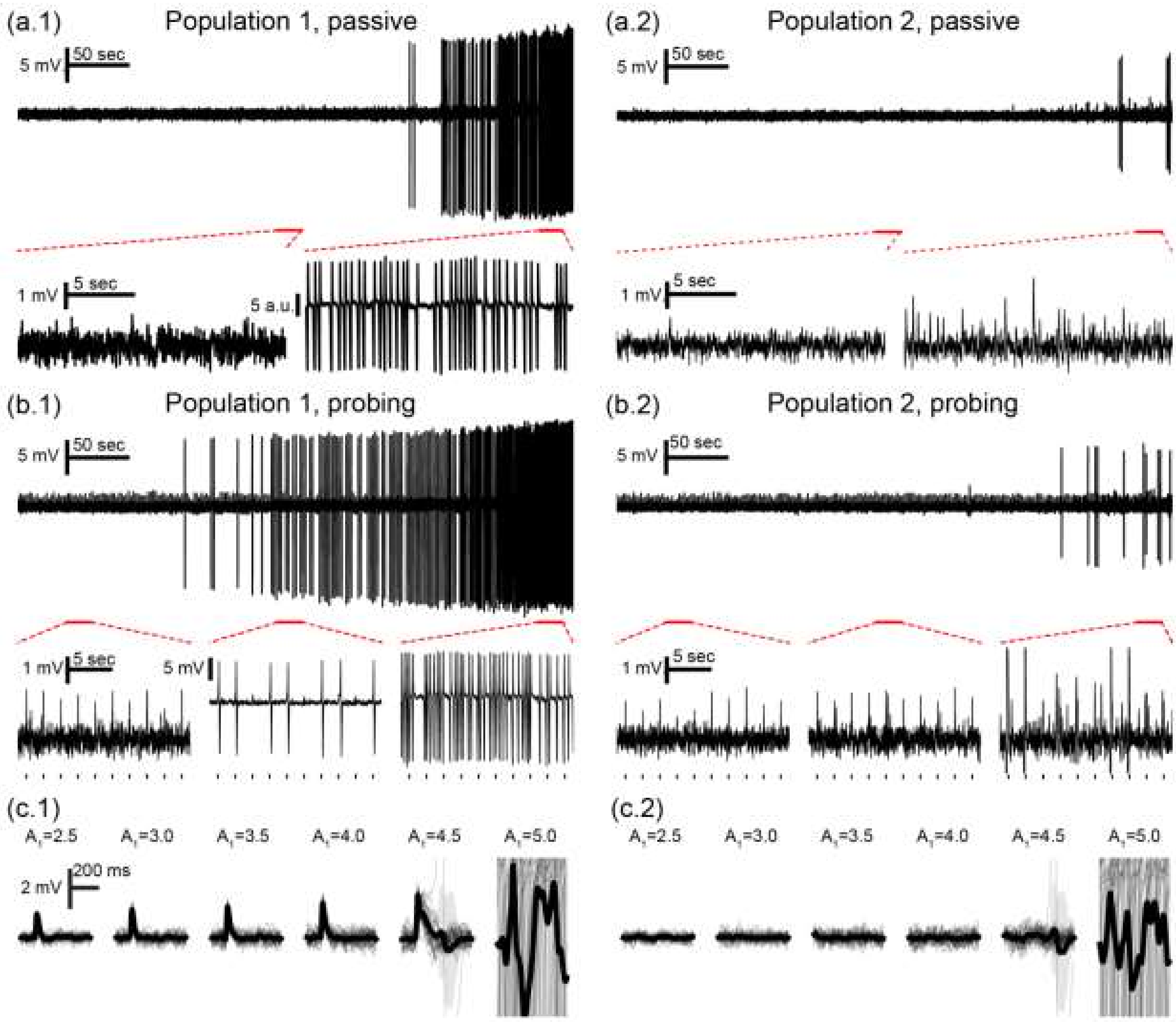

7. Setting III-A is mentioned in ln. 378. It is not described in the Model setting section.

Response: We thank the reviewer for pointing this out. This should be II-A and II-B and is now corrected.
